# Chromosomal Microarray Testing in NEC: A Case Report 

**DOI:** 10.21699/jns.v5i3.338

**Published:** 2016-07-03

**Authors:** Sathyaprasad C Burjonrappa, David Schwartzberg

**Affiliations:** 1 Attending Pediatric Surgeon, Montefiore Medical Center, USA; 2 New York University Medical Center, USA

**Keywords:** Chromosomal Micro Array, NEC, Testing

## Abstract

Necrotizing enterocolitis (NEC) remains the most common reason for emergent surgery in the neonatal intensive care unit. The common pathophysiology in all NEC involves alteration in gut microflora, abnormal blood supply to the intestine, and uncontrolled cytokine release. We report a full-term neonate who developed NEC. The neonate had surgical resection of approximately 120cms of bowel. After an initial proximal jejunostomy she underwent a successful jejuno-ileal anastomosis with preservation of her ileocolic valve at 6 weeks of age. A little more than one year of age, she is being weaned off her parenteral nutrition (PN) as her bowel adaptation continues. A chromosomal microarray analysis (CMA) resulted in the identification of a 15q13.3 microdeletion.

## INTRODUCTION

Necrotizing enterocolitis (NEC) is a common and morbid condition largely affecting the premature infant population [1]. NEC in full-term neonates has been reported in increasing numbers and most of the affected have significant co-morbidities [2]. NEC has been variously attributed to disorders of intestinal perfusion, motility disorders, antibiotic exposure, alteration in intestinal flora, and abnormal cytokine release [1,3-6]. CMA has become the gold standard in diagnosing infantile chromosomal abnormalities even with a normal karyotyping [7]. As technology improves, microarray data allows us to characterize in genetic terms the clinical phenotypes we encounter. We report a chromosome 15q13.3 deletion after microarray testing in this full-term neonate who developed NEC.

## CASE REPORT

A baby girl was born at 39 weeks via normal spontaneous delivery to a 26-year old G2P0 who presented in labor and was Group B streptococcus (GBS) positive by record. The mother had artificial rupture of membranes approximately one hour prior to delivery with meconium stained amniotic fluid. Full-term female weighed 2700grams and was crying. There was adequate respiratory effort, however with poor tone and color. APGAR scores were 6, 8 at 1 and 5 minutes respectively. 

After birth, she remained persistently hypoglycemic and was transferred to the Neonatal Intensive Care Unit (NICU) for management of hypoglycemia and possible sepsis. Empirical antibiotic treatment was begun at this time. Further physical examination revealed a sacral dimple, newborn jaundice, and bilateral congenital hip dislocation. The patient was given ampicillin/gentamicin over a 48-hour period during which time sepsis was ruled out. An abnormal abdominal x-ray was obtained and surgery was consulted for evaluation of NEC. The patient was slow to gain weight in the NICU and had difficulty in tolerating oral or gavage feeds. Due to episodes of bilious aspirate, the patient underwent an upper gastro-intestinal (UGI) study on 10th day of life which showed a dilated duodenum and dilated loops of bowel with no evidence of malrotation. On day of life 22, the patient appeared mottled, and had a distended abdomen. Plain films suggested portal venous gas, as well as pneumatosis. At the operation, approximately 120cm of small bowel was necrotic. Approximately 10cm of jejunum distal to the ligament of treitz was left intact as well as approximately 10cm of ileum with a competent ileocecal valve and colon. A jejunostomy and ileostomy were created. 

After surgery the patient was briefly started on trophic feeds but was never fed much orally. On day of life 45, the patient underwent G-tube placement and closure of her stoma. During her extensive hospital stay, the patient was found to be hypothyroid. Additionally, a CMA yielded a 123kB deletion in the region of the 15q13.3. The patient is currently one year old and is tolerating a greater amount of her energy intake orally and is being weaned off her TPN.

## DISCUSSION

The risk factors associated with NEC for premature infants maybe different from the risks associated for full-term infants. A high degree of suspicion for other pathologies prompted a genetic microarray revealing a 15q13.3 deletion. This is, to our knowledge, the first reported case of a 15q13.3 deletion associated with NEC. Chromosome microarray analysis (CMA) has largely replaced conventional chromosome analysis in the selected patient populations of infants with birth defects or intellectual impairments [8]. Ultrasound structural abnormalities in-utero often mandate CMA testing as loss of heterozygosity, chimerism, mosaicism and uniparental disomy can be detected by this method even when standard karyotyping is unremarkable [7,8]. Furthermore, CMA can detect abnormalities in 5-15% of patients who have had a normal Giemsa-band [7]. Frequently tested in neurological disorders such as schizophrenia and autism, chromosome 15 specifically has been identified as harboring numerous pathologies, such as Prader-Willi, Angelman syndrome, limb-girdle muscular dystrophy, Marfan syndrome and Tay-Sachs disease [7]. Subtle genetic anomalies may be the cause for NEC in the absence of gross phenotypic alterations as seen in our neonate.

Advances in early identification and risk stratification by analyzing genetics, endoplasmic reticulum stress, protein response, and immune signaling pathways have improved our understanding of the pathophysiology of NEC however we are yet to evolve management strategies that incorporate this knowledge into therapeutic strategies [4,5,9,10]. It is important to consider CMA in cases of NEC so as to counsel families of potential risk in future pregnancies. Determination of care strategies can be made with an awareness of the implications of the genetic anomaly. If we could accurately define, by CMA, the genetic abnormalities associated with NEC, potential genetic treatment strategies could be developed. This, of course, will need a large cohort of patients to be tested to map all potential genetic sites. With the multitude of anatomic (gut barrier), and physiological (intestinal microflora, microvasculature, complement and clotting cascades, and immune system) abnormalities in NEC it would not be surprising if we do find a common genetic basis predisposing the neonate to this devastating condition.

## Footnotes

**Source of Support:** Nil

**Conflict of Interest:** Nil
